# On-line analysis and in situ pH monitoring of mixed acid fermentation by *Escherichia coli* using combined FTIR and Raman techniques

**DOI:** 10.1007/s00216-020-02865-5

**Published:** 2020-08-14

**Authors:** George D. Metcalfe, Thomas W. Smith, Michael Hippler

**Affiliations:** 1 Department of Chemistry, University of Sheffield, Sheffield, S3 7HF UK; 2 Water and Environmental Engineering Group, Faculty of Engineering and Physical Sciences, University of Southampton, Southampton, SO17 1BJ UK

**Keywords:** Bioanalytical methods, Biological samples, IR spectroscopy/Raman spectroscopy, pH measurements, Biosensors

## Abstract

We introduce an experimental setup allowing continuous monitoring of bacterial fermentation processes by simultaneous optical density (OD) measurements, long-path FTIR headspace monitoring of CO_2_, acetaldehyde and ethanol, and liquid Raman spectroscopy of acetate, formate, and phosphate anions, without sampling. We discuss which spectral features are best suited for detection, and how to obtain partial pressures and concentrations by integrations and least squares fitting of spectral features. Noise equivalent detection limits are about 2.6 mM for acetate and 3.6 mM for formate at 5 min integration time, improving to 0.75 mM for acetate and 1.0 mM for formate at 1 h integration. The analytical range extends to at least 1 M with a standard deviation of percentage error of about 8%. The measurement of the anions of the phosphate buffer allows the spectroscopic, in situ determination of the pH of the bacterial suspension via a modified Henderson-Hasselbalch equation in the 6–8 pH range with an accuracy better than 0.1. The 4 m White cell FTIR measurements provide noise equivalent detection limits of 0.21 μbar for acetaldehyde and 0.26 μbar for ethanol in the gas phase, corresponding to 3.2 μM acetaldehyde and 22 μM ethanol in solution, using Henry’s law. The analytical dynamic range exceeds 1 mbar ethanol corresponding to 85 mM in solution. As an application example, the mixed acid fermentation of *Escherichia coli* is studied. The production of CO_2_, ethanol, acetaldehyde, acids such as formate and acetate, and the changes in pH are discussed in the context of the mixed acid fermentation pathways. Formate decomposition into CO_2_ and H_2_ is found to be governed by a zeroth-order kinetic rate law, showing that adding exogenous formate to a bioreactor with *E*. *coli* is expected to have no beneficial effect on the rate of formate decomposition and biohydrogen production.

**Electronic supplementary material:**

The online version of this article (10.1007/s00216-020-02865-5) contains supplementary material, which is available to authorized users.

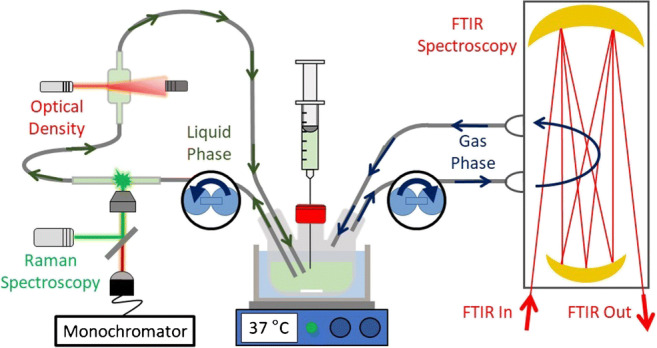

## Introduction

Microbial fermentation is a fascinating and important process with broad relevance to the fields of biotechnology, environmental science, and medicine. In the absence of terminal electron acceptors, such as oxygen, various forms of fermentation are carried out by many facultative and obligate anaerobes in order to obtain energy to sustain growth and cellular functions [1]. The production of organic compounds characterises all microbial fermentations; in some cases, these products can be valuable chemical intermediates or commodities such as with acetone-butanol-ethanol (ABE) fermentations [2] or the brewing process carried out by *Saccharomyces cerevisiae* [3].

**Fig. 1:**
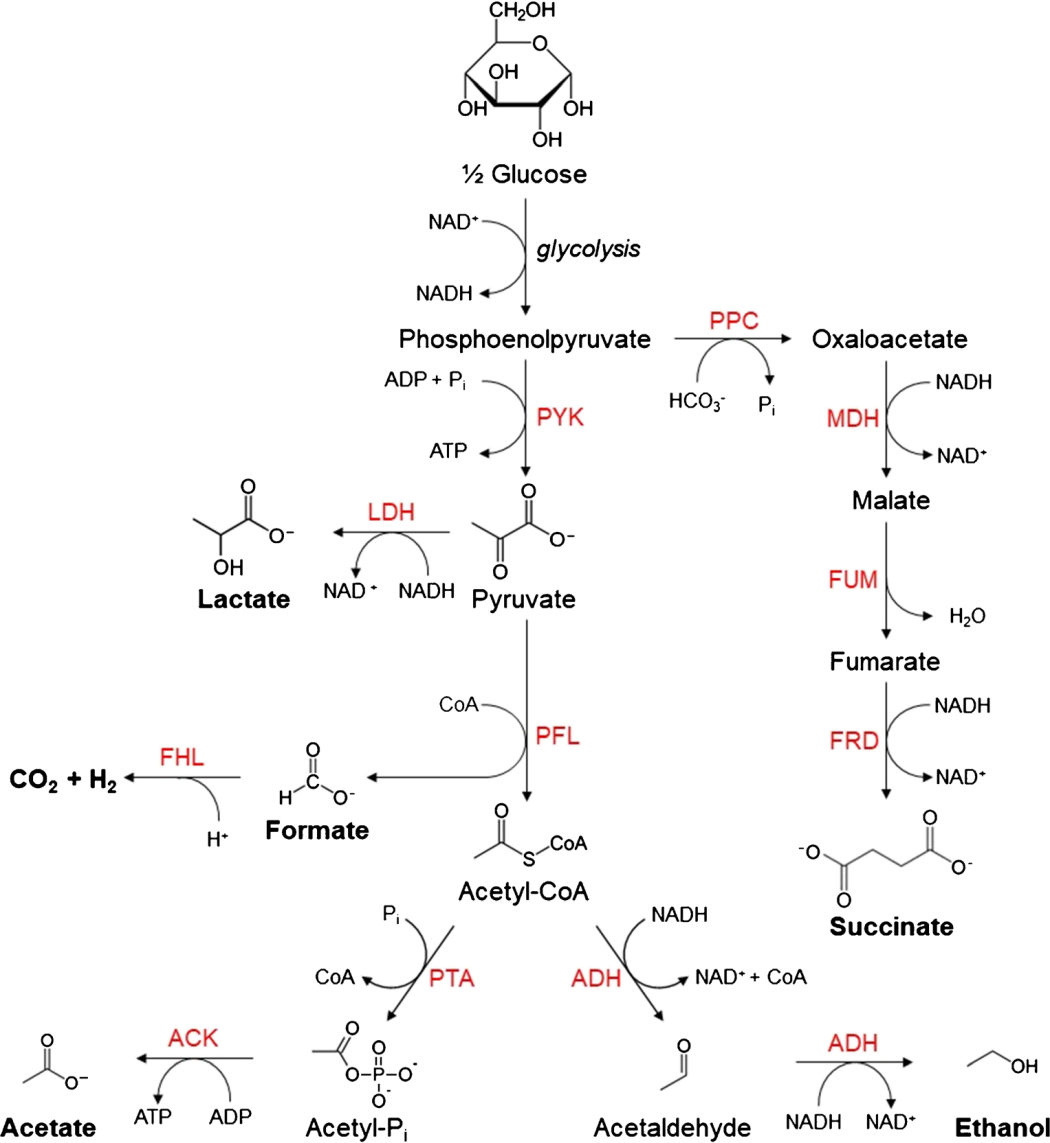
*E*. *coli* mixed acid fermentation pathways with glucose as a carbon source. End products are shown in boldface. The enzymes responsible for each step are indicated in red: ACK, acetate kinase; ADH, alcohol dehydrogenase; FHL, formate hydrogenlyase; FRD, fumarate reductase; LDH, lactate dehydrogenase; MDH, malate dehydrogenase; PFL, pyruvate formatelyase; PPC, phosphoenolpyruvate carboxylase; PTA, phosphotransacetylase; PYK, pyruvate kinase

*Escherichia coli* (*E. coli*) has proven to be a particularly useful platform in biotechnology due to its relatively well-understood biochemistry. *E*. *coli* utilises a mixed acid fermentation when grown anaerobically with glucose (see scheme in Fig. 1) with up to one-third of carbon derived from glucose converted to formate [1, 4]. Above an external pH of 7, the major fermentation products are acetate, ethanol, and formate [5]. Acidic products are excreted to prevent cytoplasmic acidification, decreasing the extracellular pH. When the external pH drops below 6.8, formate is reimported back into the cell to be disproportionated to CO_2_ and H_2_ by the formate hydrogenlyase (FHL) complex [6–9]. Two additional hydrogenases are also expressed, which can then reoxidise H_2_ [10, 11]. As an additional strategy during later fermentation stages, *E*. *coli* switches to lactate instead of acetate and formate production to decrease further acidification [5, 12]. Given its wide range of fermentation products, *E*. *coli* has been investigated as a potential platform for biologically produced biohydrogen, bioethanol, succinic acid, and biopolymers such as polylactic acid (PLA) [13, 14]. As FHL activity to produce H_2_ is dependent on the pH, it is a critical control parameter in the conversion of organic feedstock to biohydrogen [8–10].

While chromatographic techniques are widely used in offline process monitoring, the limited throughput and lengthy analysis times associated with these techniques make them unsuitable for continuous monitoring [15]. Electrochemical sensors, in contrast, are compact and cheap and can be easily integrated into process control systems, enabling continuous on-line analysis and control. They are widely used for monitoring pH, the concentration of various ions, and dissolved oxygen. Despite their widespread use in bioprocesses, they require frequent servicing and are susceptible to cross-interferences from other chemical species, to changes in solution activity and long-term drift. Submerging sensors into a bioreactor also increases contamination risk, which is a problem shared with all invasive techniques [15].

Spectroscopic techniques are powerful analytical tools which allow for rapid, selective, and on-line measurements. The non-invasive nature of using light to probe a bacterial culture in a closed system also enables greater confidence in preventing contamination. FTIR and other techniques using mid-infrared wavelengths are sensitive as they rely on strong fundamental vibrational transitions. While they do find some application, their usefulness is limited in solution to some extent by the broad absorption profile of water over almost the entire region. In a couple of previous studies, liquid-phase FTIR has been applied to monitor bioprocesses, usually employing an attenuated total reflection (ATR) probe immersed in the suspension. Examples include monitoring glucose and lactic acid in *Lactobacillus casei* fermentation [16], glucose and acetate in *E*. *coli* fermentation [17], or the fermentation of phenoxyacetic acid to penicillin V by *Penicillium chrysogenum* [18]. Compounds can be detected at millimolar levels, but the probes require sterilisation before use which introduces issues with disassembling the equipment and maintaining optical performance. In contrast, in the vapour phase, the lack of complex hydrogen-bonded networks of water molecules leads to narrower and better defined absorption bands, with windows which are relatively free of water vapour, enabling sensitive detection of volatile organics and oxygen-containing compounds without interference from water. Using Henry’s law, the partial pressures of volatiles and gaseous products determined in the headspace can be converted to their dissolved concentrations (see also refs. [11, 19, 20]).

For direct analysis of the liquid phase, near-infrared (NIR) spectroscopy is much more widely applied in bioprocess and fermentation monitoring [21–24] due to the much weaker absorption profile of water in this region. However, the spectral features observed in NIR spectroscopy are overtone or combination transitions that are also weaker, typically by at least an order of magnitude compared with the corresponding fundamental transitions, reducing the inherent sensitivity. Also, such overtone bands are broad and typically less characteristic with significant overlap of spectral features from different compounds, leading to the use of complex mathematical models which are not easily transferable between different systems or media types. In a recent comparison of NIR with ATR-FTIR monitoring of fermentation processes, NIR has been found to have high prediction errors due to light scattering and the lack of distinctive and sharp spectral features [16].

Raman spectroscopy is a complementary technique to absorption-based techniques; like NIR spectroscopy, Raman spectroscopy is comparatively insensitive towards water although bands are typically far more characteristic of the compounds being measured. Raman spectroscopy has seen extensive applications in biotechnology [25–27], including in lignocellulosic fermentation for bioethanol production [28]. In most applications, a Raman probe is inserted into the suspension which introduces problems maintaining sterile conditions, optical performance and disassembling the reactor for sterilisation before use. As an example, in a previous Raman application to monitor glucose, acetate, formate, lactate, and phenylalanine in the aerobic metabolism of *E*. *coli*, changes in optical characteristics of the probe and its sapphire window after steam sterilisation have been reported [25]. Although theoretical detection limits of the order of 0.1 mM at 300 s integration have been predicted to be possible, sensitivity is ultimately limited and compromised due to systematic errors caused by spectral shifts between reference and sample spectra [25]. Severe interferences are possible in Raman spectroscopy due to fluorescence, in particular, if growth media like lysogeny broth (LB) are used that are coloured and have visible absorption bands [29]. This limitation requires careful design and selection of excitation wavelength, as discussed below in the ‘Results and discussion’ section. Raman spectroscopy itself is not a very sensitive technique due to low scattering cross sections. One technique to overcome sensitivity issues is to apply resonance Raman spectroscopy; this requires analytes having absorption bands close to the excitation wavelength, but not overpowering Raman signals by fluorescence. This condition, however, is only achieved in special cases, like carotenoid detection at micromolar levels in yeast fermentation at 785 nm Raman excitation [30]. In addition to applications in process monitoring, vibrational spectroscopies are powerful emerging tools for in situ bacterial species identification [31].

Here we describe and characterise the performance of a combined non-invasive FTIR and Raman approach in the gas phase and solution, respectively, for monitoring and controlling microbial fermentation, with the mixed acid fermentation of *E*. *coli* serving as a particularly relevant example. We demonstrate that Raman monitoring of the two major phosphate species present in the growth medium allows the spectroscopic determination of the pH of the solution, an important parameter in fermentation processes, and we derive the theoretical basis of this analysis. Critical issues associated with electrochemical probes, including contamination and long-term drift, are avoided by being able to measure pH in situ accurately and on-line. Combined with continuous optical density (OD) measurements, a more conventional measure of cellular activity, we are finally able to obtain interesting insights into the metabolism of the cell and the production of acids, ethanol, and formate disproportionation.

## Experimental

**Fig. 2:**
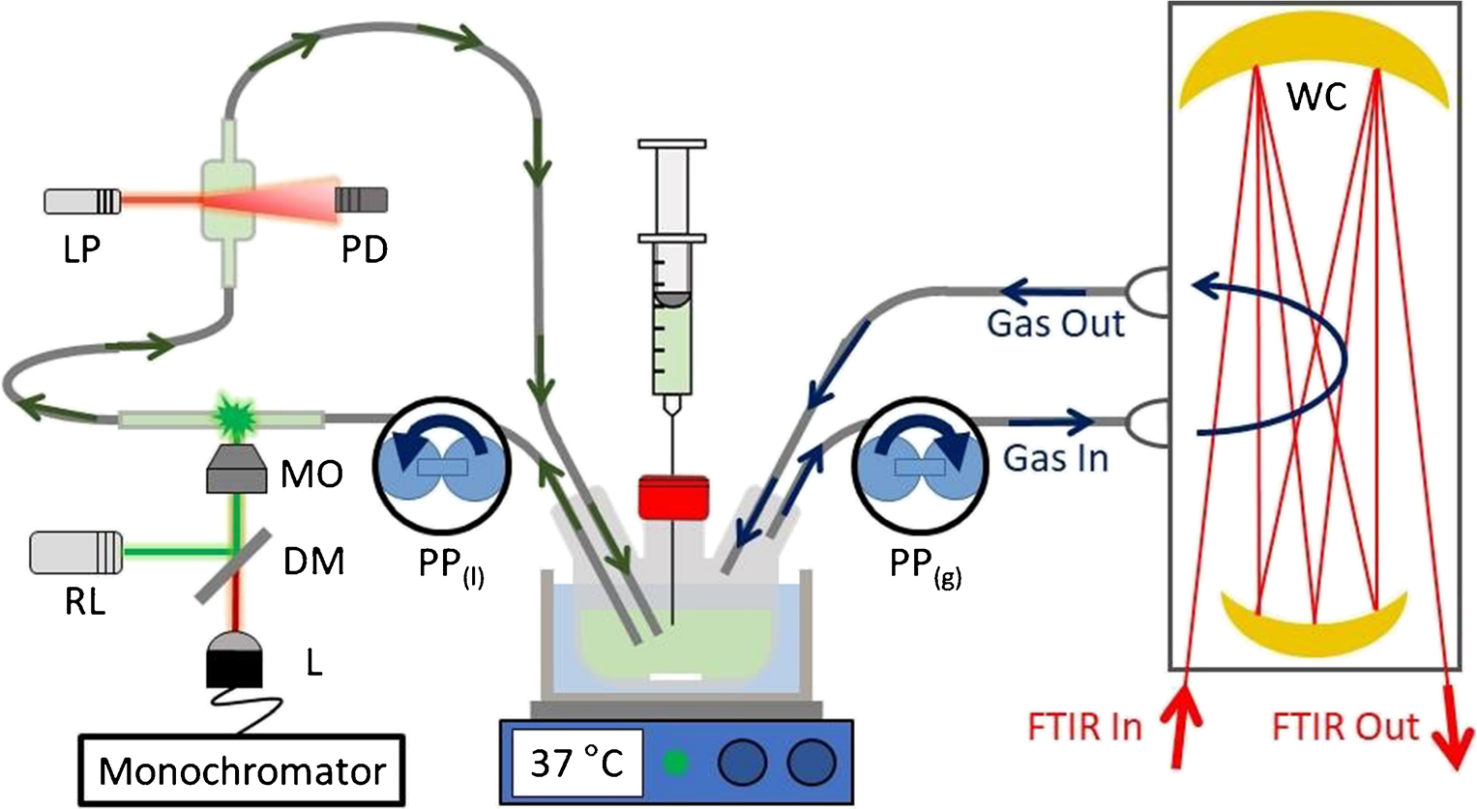
Scheme of the experimental setup for spectroscopic analysis of bacterial fermentation. DM, dichroic mirror; L, lens; LP, laser pointer; MO, microscope objective; PD, photodiode; PP_(g)_, gas-phase peristaltic pump; PP_(l)_, liquid-phase peristaltic pump; RL, Raman laser; WC, White cell

Figure 2 shows a scheme of our experimental setup. About 60 mL of a bacterial suspension at 1 bar is contained in a 37 °C thermostatted and stirred 5-neck custom flask from where the suspension and headspace are each cycled using peristaltic pumps (PP, 3 L/h). From the left-neck, the bacterial suspension is cycled for in situ OD_600_ (optical density at 600 nm in a 1-cm cuvette) and liquid Raman measurements. From the right-neck, the headspace is cycled for gas-phase FTIR measurements in a long-path White cell (WC). The middle-neck is equipped with a rubber septum enabling reagent addition and sampling of the culture for gas chromatography (GC) analysis and external pH measurements. Not shown in Fig. 2 are two further ports, one leading to a vacuum pump and the other connecting to a N_2_ cylinder. These vacuum and N_2_ lines enable purging of O_2_ from the sealed airtight system to give anaerobic growth conditions. The total headspace volume is 525 mL.

Production of acetaldehyde, ethanol, and CO_2_ was determined by gas-phase FTIR spectroscopy taking recordings continuously every 5 min. To detect the trace quantities of the volatile fermentation products, a Mattson Research Series FTIR instrument (0.4 cm^−1^ spectral resolution, no apodisation, liquid N_2_–cooled MCT detector) is used with a home-built multiple-pass absorption White cell equipped with CaF_2_ windows and 2-in. (50.4 mm) gold mirrors. The White cell can be aligned to provide 20–40 passes (in steps of 4) at a base length of 20 cm, affording a total path length of 4–8 m; in the present configuration, the path length is set to 4.0 m as confirmed by calibration with water lines. The White cell was heated to about 45 °C by heating tape to avoid water condensation. The spectral range of the FTIR instrument is from 1000 to 7000 cm^−1^, limited at lower wavenumbers by the CaF_2_ windows and higher wavenumbers by the globar light source. Acetaldehyde and ethanol partial pressures were determined by FTIR spectroscopy using the Beer-Lambert law and reference spectra; partial pressures can then be converted into concentration in the solution using Henry’s law (see also refs. [11, 19, 20]).

The liquid-phase peristaltic pump (Fig. 2, PP_(l)_) circulated the bacterial suspension from the custom flask to a sealed borosilicate tube and then a 1-cm glass cuvette before returning to the flask. This circuit for the bacterial suspension had a volume of approximately 10 mL. Red light from a laser pointer (LP, 1 mW, 650 nm) was scattered by the bacterial suspension flowing through the 1-cm glass cuvette to measure the OD_600_ in situ. The transmitted intensity was observed by a photodiode (PD) and calibrated with start and end-point OD_600_ values externally measured using a UV-Vis spectrometer. Typical end-point OD_600_ values for the bacterial suspension were around 1.0–1.2. The bacterial suspension flowing through the sealed borosilicate tube was analysed by liquid-phase Raman spectroscopy using a home-built spectrometer. Briefly, a 532.2-nm, 20-mW cw laser (Lasos, GL3dT), denoted as Raman laser (RL) in Fig. 2, is turned by 90° by a small mirror (DM) and coupled into a microscope objective (MO). The microscope objective focused the green laser-light into the glass tube as well as collimating the resulting Raman backscattered light. The backscattered light passed through DM and was coupled into a lens (L) and transmitted to the monochromator (Shamrock SR-750-A) equipped with a 1200-L/mm grating and CCD camera (Andor i-Dus DU420A-OE at − 80 °C). The grating provided a 880 cm^−1^ spectral range from 900 to 1780 cm^−1^ at about 0.8 cm^−1^ resolution. After wavenumber calibration, Raman peak position accuracy is estimated to be ± 3 cm^−1^ (for more details, see refs. [32–35] and the Electronic Supplementary Material (ESM)). Raman spectra were recorded every 5 min in 10 accumulations of 30 s.

The bacterial suspension was also sampled during growth for GC analysis of acetate and ethanol to compare with the liquid-phase Raman and gas-phase FTIR methods, respectively. 0.2 mL of sample was dissolved in 0.8 mL acetone, and 0.3 μL of this solution injected into a standard GC instrument (temperature programmed Agilent DB-WAX UI, with 1.4 mL/min H_2_ carrier gas and flame ionisation detector). From the GC peak integral, the concentration of the sample was determined after a calibration. For comparison with the in situ spectroscopic pH measurements, start and end-point samples were taken and the pH recorded externally using a Mettler Toledo SevenMulti pH meter.

*E*. *coli* (strain K-12 MG1655) was transferred from glycerol stock (maintained at − 80 °C) and streaked on sterile, LB agar plates (LB, lysogeny broth, a nutrient-rich growth medium). Plates were left overnight at 37 °C to allow the growth of distinct single colonies. Before each measurement, 50 mL of sterile LB medium was inoculated with a single *E*. *coli* colony and incubated anaerobically in a sealed 50-mL centrifuge tube for 16 h (37 °C, 200 rpm) to a typical OD_600_ of 2.0. A total of 1 mL of the starter culture was centrifuged to remove the LB and resuspended into 1 mL of fresh, sterile M9 minimal medium. The fresh M9 medium contained 30 mM glucose, 47 mM Na_2_HPO_4_, 22 mM KH_2_PO_4,_ 8.5 mM NaCl, 18 mM NH_4_Cl, 1 mM MgSO_4_, 0.3 mM CaCl_2_, 68 nM biotin, 63 nM thiamine, 1 μM Na_2_MoO_4_·2H_2_O, 1.7 μM Na_2_SeO_3_, and other standard trace elements. A further 60 mL of M9 medium was prepared in the 5-neck custom flask. The flask was prewarmed and maintained at 37 °C using a thermostated water bath under rapid stirring for efficient gas transfer. Monitoring of fermentation began with the injection of the 1 mL M9 medium containing the *E*. *coli* into the 60 mL fresh M9 medium in the flask giving a typical starting OD_600_ of 0.03. Some experiments had a further injection of 1 mL potassium formate (final concentration 40 mM) 3 h after addition of the *E*. *coli*. At the end of fermentation, the bacterial suspension was centrifuged, washed, and dried in order to record the dry biomass (typically around 30 mg).

## Results and discussion

### Spectroscopic analysis of microbial fermentation processes

#### Liquid Raman spectroscopy for phosphate, formate, and acetate analysis

**Fig. 3:**
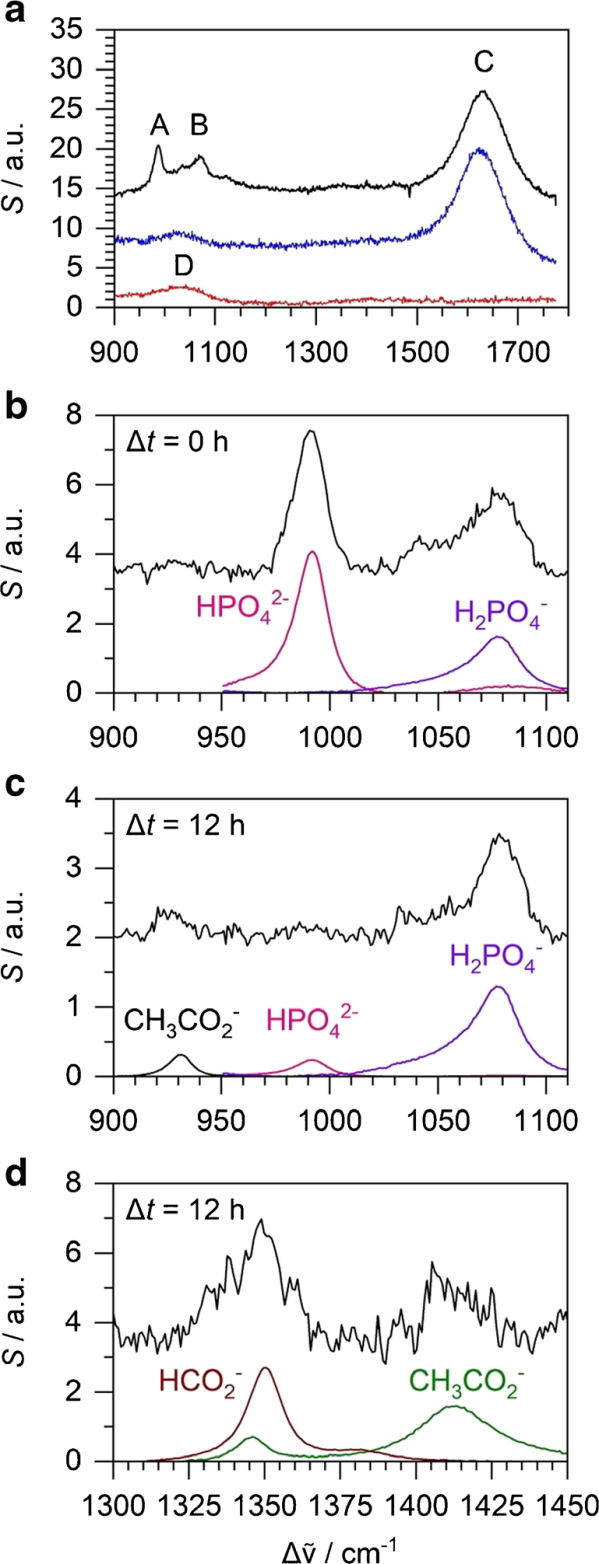
Experimental Raman spectra. **a** Fresh M9 medium (upper trace, black), water (middle trace, blue), and empty glass tube (lower trace, red). **b** Bacterial suspension at the start of anaerobic fermentation with peaks due to 47 mM HPO_4_^2−^ and 22 mM H_2_PO_4_^−^. **c** After 12 h of anaerobic fermentation with peaks due to 3 mM HPO_4_^2−^, 66 mM H_2_PO_4_^−^, and 20 mM acetate. **d** After 12 h of anaerobic fermentation with peaks due to 20 mM acetate and 15 mM formate

With our setup, liquid Raman spectra are obtained within 900–1780 cm^−1^ which covers characteristic phosphate (HPO_4_^2−^, H_2_PO_4_^−^), formate, acetate, and water peaks in the bacterial suspension. Raman scattering by the acids and other metabolites within the bacterial cells likely also contributes to the experimental spectra. However, as the sum volume of bacterial cells is much less than the volume of the liquid medium, it is assumed the experimental spectra correspond mainly to the species in solution. For comparison and calibration, Raman reference spectra were obtained in borosilicate NMR tubes. Raman spectra of the phosphate-buffered M9 medium (47 mM monobasic and 22 mM dibasic potassium phosphate), water, and the empty tube as references are shown in Fig. 3a. Characteristic features are peak A of HPO_4_^2−^ at 989 cm^−1^ and peak B of H_2_PO_4_^−^ at 1076 cm^−1^; outside the range displayed, H_2_PO_4_^−^ also has an additional peak of similar intensity at 876 cm^−1^ [36]. Peak C is the bending vibration of the water solvent at 1630 cm^−1^. Peak D is a silicate peak of the borosilicate tube; this peak is subtracted as an artefact from experimental Raman spectra, as shown in Fig. 3b and c. Raman intensities were normalised using the water peak; this normalisation is particularly relevant for our biological samples which become turbid with time. In the normalisation, the water peak is fitted by a Gaussian contour centred at 1630 cm^−1^ with FWHM of 80 cm^−1^. Normalisation assumes that the area of this Gaussian is the same in all solution Raman spectra because water concentrations remain the same. Reference spectra of 1 M Na_2_HPO_4_ and KH_2_PO_4_ phosphate solutions were recorded for calibration to obtain the concentration of phosphate anions from Raman spectra. A self-written computer programme implements a least squares fit of the 950–1110 cm^−1^ region of an experimental Raman spectrum to the sum of the reference spectra of the two phosphate anions and a linear baseline (see Fig. 3b). The multipliers of the reference spectra are then converted into concentration. Comparing reference solutions with known mixed phosphate concentrations ranging from 10 to 500 mM, concentrations measured with Raman spectroscopy show excellent linearity and are estimated to be accurate within 10% (linear regression coefficient *R* = 0.9997, standard deviation of percentage error 8.1%).

Figure 3b, c, and d show Raman spectra obtained at different stages during the anaerobic fermentation of *E*. *coli* (see ‘Mixed acid fermentation of *E*. *coli*’ for more details). In addition to the phosphate peaks, there were characteristic peaks from acetate [37, 38] (at 928 and 1414 cm^−1^) and formate [37] (at 1349 cm^−1^). Reference spectra of 1 M ammonium acetate and potassium formate standards were also recorded as calibration to obtain formate and acetate concentrations of experimental Raman spectra. In the fitting procedure, the 1310–1450 cm^−1^ region is fitted to the sum of formate and acetate references and a linear baseline (see Fig. 3d). Recording a series of Raman spectra (as in ‘Mixed acid fermentation of *E*. *coli*’) and analysing the noise level (standard deviation) of the baseline (essentially a blank M9 sample) provides an estimate for the noise equivalent detection limit of this method. With a standard signal integration time of 5 min, this detection limit would be about 2.6 mM for acetate and 3.6 mM for formate. With additional averaging to an integration time of 1 h, the limits improve to 0.75 mM for acetate and 1.0 mM for formate. Note that this is the noise equivalent detection limit (1 σ limit); practical detection limits are often quoted as 3 σ in the literature. The analytical range extends to at least 1 M, and we expect a similar standard deviation of percentage error as in the case of the phosphate ions, i.e., about 8%. This level of sensitivity and time resolution is adequate for the fermentation experiment introduced below, where acetate and formate concentrations up to 20 mM have been measured in a continuous fermentation lasting up to 60 h.

Two main factors are limiting the analytical performance of liquid Raman spectroscopy in biological samples. First, with increasing cell density, the solution becomes more and more turbid and reduces Raman signal intensity. Increased turbidity seriously affects sensitivity and, more importantly, calibration. Calibration can be maintained by normalisation, either using the OD value or, as discussed above, using the water bending vibration as a reference Raman peak. Another factor which often plagues Raman spectroscopy is interference by fluorescence which can mask Raman signals. Fluorescence was not an issue in our biological experiments where we have used M9 minimal medium which is colourless and non-fluorescent, and where the metabolites are also non-fluorescent. It has been shown before, however, that this can become limiting if coloured growth media like LB are used [29]. Fluorescence can often be alleviated by moving to a longer wavelength Raman excitation, towards the red or near IR.

**Fig. 4:**
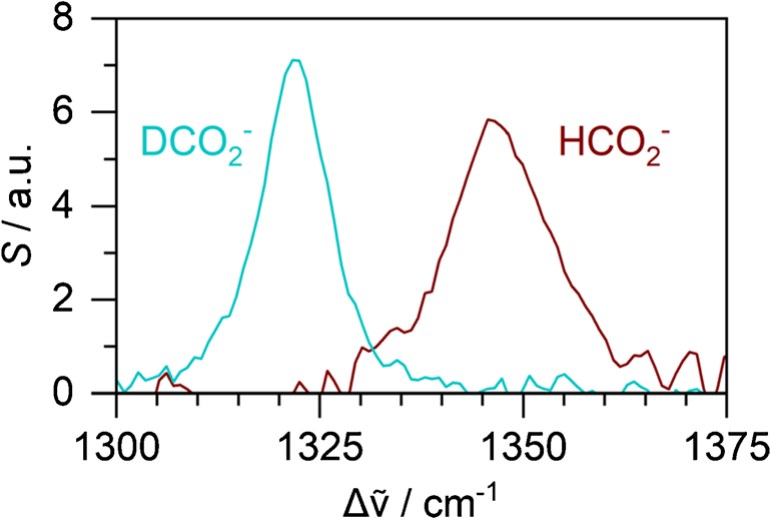
Raman spectra of 40 mM deuterated (blue) and unlabelled (red) formate solutions

To demonstrate the potential of liquid-phase Raman spectroscopy for future isotope labelling experiments, we have also recorded reference spectra of unlabelled and deuterated formate (see Fig. 4). The deuteration shifts the 1349 cm^−1^ band by ca. 27 cm^−1^ towards lower wavenumbers. Both isotopomers are separated and distinguishable from each other by Raman spectroscopy. Future work will include studying microbial formation and disproportionation of isotopically labelled formate.

#### In situ pH measurements by Raman spectroscopy

In situ pH was calculated using the detected concentrations of the phosphate anions HPO_4_^2−^ and H_2_PO_4_^−^ which act as a buffer in M9 medium. In a phosphate buffer around pH = 7, the relevant acid dissociation reaction is:

1 $$ {\mathrm{H}}_2{{\mathrm{PO}}_4}^{-}\ \left(\mathrm{aq}\right)={\mathrm{H}}^{+}\ \left(\mathrm{aq}\right)+{{\mathrm{H}\mathrm{PO}}_4}^{2-}\ \left(\mathrm{aq}\right) $$

with p*K*_a,2_ = 7.20 at 25 °C [39]. A modified Henderson-Hasselbalch equation gives the pH of this buffer solution (derived in ref. [40] demonstrating that activities have to be used instead of actual concentrations for accurate results):2where *a* = *ɣ c* are activities, *ɣ* activity coefficients, and *c* the actual concentrations. The *ɣ* depends mainly on the ionic strength *I* of the solution. They can be approximated by the Debye-Hückel theory for highly diluted electrolytes or, more appropriate in the present case, by the Davies modification extending the range to higher *I* (up to ≈ 0.5) [40–43]. The standard form of the Henderson-Hasselbalch equation uses actual concentrations, not activities, which would lead to errors over 0.3 for pH determinations in our application. We have found that modified Eq. (2) including the Davies correction for activities can provide pH values in the 6–8 pH range with an accuracy better than 0.1 [40]. This allows the accurate determination of the pH of a phosphate buffer solution in situ, without sampling, by Raman spectroscopy, for example, of a bacterial culture. pH measurement by Raman spectroscopy has been demonstrated before, for example, to analyse meat samples (but not in the context of monitoring bacterial suspensions, to our knowledge) [44, 45]. The undefined nature of (meat) samples and the use of the original, unmodified Henderson-Hasselbalch equation, however, introduced inaccuracies, and therefore, spectroscopic pH determination is most often done by an empirical, statistical principal component or regression analysis to relate certain spectral changes to pH. In our application, the defined M9 medium with phosphate buffer allows the direct observation of the concentration of an acid and its base, which is related to pH via modified Eq. (2). An additional distinct advantage of our approach is that the shift of HPO_4_^2−^ concentration towards H_2_PO_4_^−^ directly reflects the absorption of H^+^ of any acids introduced to the phosphate buffer [40]. That means that the change in concentration of phosphate anions as observed by Raman spectroscopy corresponds to the total net concentration of acid H^+^ introduced or produced in situ which is very relevant in monitoring mixed acid fermentation of microbes (see ‘Mixed acid fermentation of *E*. *coli*’).

#### White cell FTIR spectroscopy for CO_2_, ethanol, and acetaldehyde analysis

**Fig. 5:**
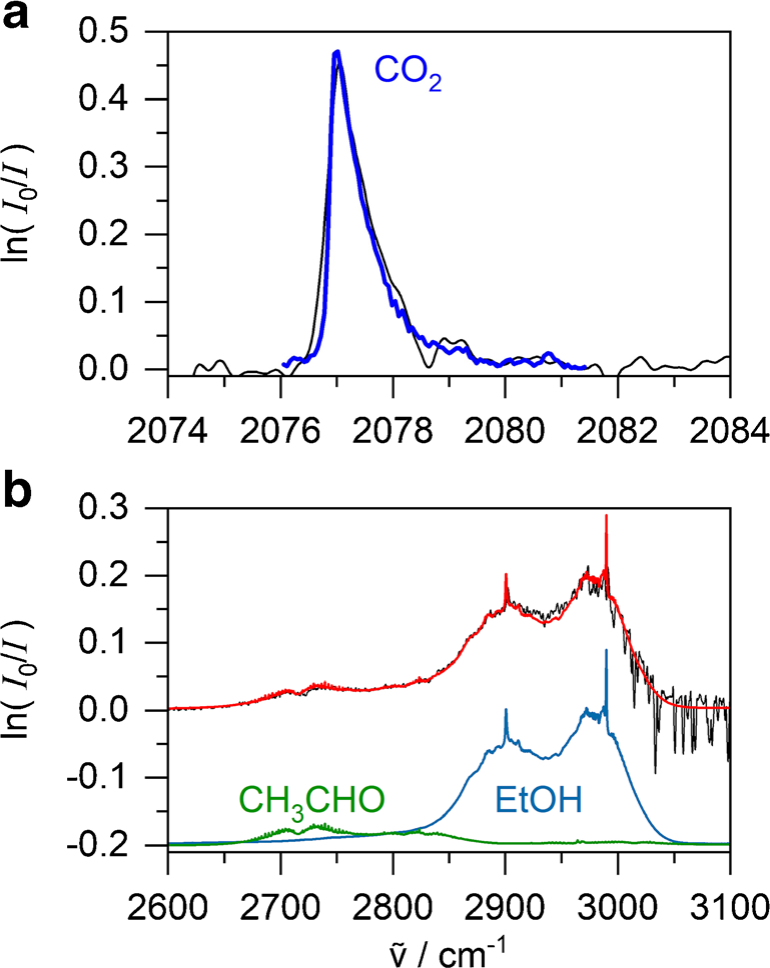
FTIR spectrum taken after 12 h of *E*. *coli* with fitted PNNL model spectra. Panel **a**: experimental CO_2_ peak corresponding to 21 mbar shown in black with fitted CO_2_ model spectrum in blue. Panel **b**: overlapping experimental bands of acetaldehyde and ethanol corresponding to 18 and 105 ppm, respectively, shown in black. Acetaldehyde and ethanol model spectra are shown in green and blue, respectively, with the sum of both models shown in red

FTIR spectra were recorded using the 4.0 m White cell from 1000 to 7000 cm^−1^ at 0.4 cm^−1^ spectral resolution to detect vapour-phase species from the metabolism of bacteria. The spectra are dominated below 2000 cm^−1^ and between 3000 and 3500 cm^−1^ by rotationally resolved water lines, and are completely saturated in the 2200–2400 cm^−1^ and 3500–4000 cm^−1^ region by the CO_2_ and water stretching fundamentals, respectively. CO_2_ is perhaps the most indicative gas of bacterial metabolism and activity. For its quantification by FTIR spectroscopy, the main CO_2_ absorption bands cannot be used due to saturation even under ambient levels. We have evaluated a weak overtone/combination band (the *ν*_1_ + *ν*_2_/3*ν*_2_ Fermi resonance near 2077 cm^−1^) and found it very suitable since it has a very distinct and characteristic sharp *Q*-branch peak (0.7 cm^−1^ FWHM) which does not suffer any noticeable interferences (see Fig. 5a). A self-written computer programme performs a linear baseline correction of experimental FTIR spectra and integrates the *Q*-branch from 2076 to 2081.5 cm^−1^. Comparison with a reference gas-phase FTIR spectrum for 1 ppmv CO_2_ from the Pacific Northwest National Laboratory (PNNL) database [46] then provides the CO_2_ partial pressure of the headspace above bacterial suspensions (at 1 bar total pressure, 1 μbar = 1 ppmv).

Careful inspection of FTIR spectra revealed two further products of bacterial metabolism which can be detected in the headspace above bacterial suspensions, acetaldehyde and ethanol. Ethanol is a very relevant end product of fermentation in bioprocesses and acetaldehyde is a precursor of its biosynthesis (see Fig. 1). Their spectral signatures are apparent in the C-H stretching region of 2575–3100 cm^−1^ (see Fig. 5b). In this case, simple integration of spectral features would not work since both spectra are overlapping and ethanol is affected by water interferences at the higher wavenumber end of its band. Here, we applied a similar routine as in the liquid Raman spectral analysis; in a fitting procedure, the 2575–3100 cm^−1^ region is fitted to the sum of acetaldehyde and ethanol PNNL reference spectra and a linear baseline (see Fig. 5b). The multipliers of the reference spectra are then converted into partial pressures.

Using Henry’s law, the molarity of a dissolved gas can be calculated from its partial pressure. This allows an estimation of molar concentrations of CO_2_, ethanol, and acetaldehyde in the bacterial suspension from the FTIR measurements in the headspace (see also refs. [11, 19, 20]). Henry’s law constants for aqueous solutions at 37 °C are calculated as 0.026 mM mbar^−1^ for CO_2_, 82 mM mbar^−1^ for ethanol, and 6.3 mM mbar^−1^ for acetaldehyde [47]. Additionally, using the ideal gas law, we estimated that 7.0% CO_2_, 99.6% ethanol, and 94.9% acetaldehyde would be held in solution. It can be seen that CO_2_ is slightly soluble in water; once it has dissolved, a small proportion of the CO_2_ reacts with water to form carbonic acid which will be at equilibrium with bicarbonate and carbonate ions, depending on the pH. Under our conditions, less than 1% of dissolved CO_2_ will be lost to carbonic acid and carbonates. The FTIR determination is in situ, on-line and allows continuous and sensitive monitoring without sampling, which is an important advantage for controlling bioprocesses. A standard FTIR measurement uses 128 accumulations and takes 2 min to acquire. An analysis of the baseline of acetaldehyde and ethanol partial pressures as determined by our fitting routine then provides a standard deviation of 0.21 μbar for acetaldehyde and 0.26 μbar for ethanol which may serve as an estimate for the noise equivalent detection limit. Using Henry’s constants, these detection limits correspond to 3.2 μM acetaldehyde and 22 μM ethanol. The analytical dynamic range exceeds 1 mbar ethanol in the gas phase corresponding to 85 mM in solution; for quantifying higher concentrations, shorter path lengths and/or other spectroscopic bands are available.

### Mixed acid fermentation of *E*. *coli*

**Fig. 6:**
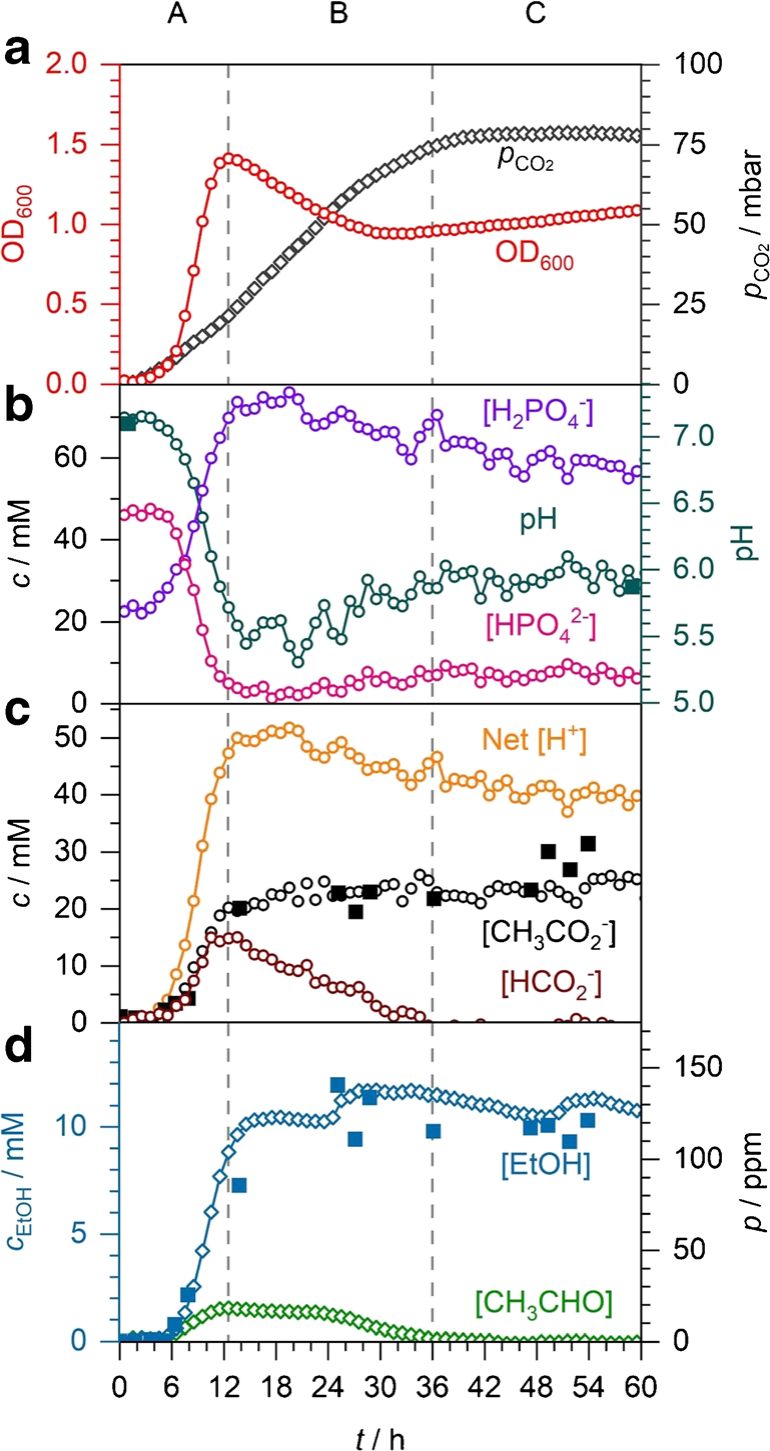
*E*. *coli* anaerobic fermentation in M9 medium. A to C denote three distinct phases: formate accumulation, decline, and depletion, respectively. **a** OD of the bacterial culture and partial pressure of CO_2_. **b** Concentrations of the phosphate buffer components with in situ, spectroscopic pH (open circles) and externally measured pH (solid squares). **c** Concentrations of acetate, formate, and net acid produced. Acetate quantification by Raman (open circles) and GC (solid squares) is compared. **d** Partial pressures of ethanol and acetaldehyde. Ethanol concentrations by FTIR (open diamonds) and GC (solid squares) are compared

In an applied example of our spectroscopic analysis, we monitored the mixed acid fermentation of *E*. *coli*. This application is particularly relevant because fermentation processes have a pronounced pH dependence and are essential in biotechnology to produce biohydrogen and other high value chemicals. On-line spectroscopic analysis of metabolites and pH enables process control and optimisation. Figure 6 shows a typical example of the time-dependent concentrations, pH, and OD measurements taken during the spectroscopic monitoring of anaerobic *E*. *coli* grown in 60 mL M9 medium supplemented by 30 mM glucose. Experiments were repeated in triplicate, and all repeats showed essentially the same qualitative and quantitative behaviour. OD is a conventional and widely employed technique to distinguish bacterial growth phases and estimate bacterial density (but also compare ref. [20]). In the experiment, the OD started quite low (ca. 0.03) and did not increase noticeably for about 3 h. This is typical for the lag phase, where the bacterial cells prime themselves for cell division. From about 3 to 12 h, the OD rises to its peak value of 1.4, indicative of the exponential growth phase. Phase A in Fig. 6 denotes both the lag and exponential growth phases. After this, the stationary phase is expected where cell death and division are at equilibrium (phase B), followed by the end of fermentation when the glucose-limited culture runs out of organic feedstock (phase C). The OD measurements are neither very specific nor conclusive in phases B and C, as the OD was observed to first fall to 0.9 from 12 to 30 h, followed by a steady, slow rise. Based on the OD alone, it is unclear what is happening; these changes could be caused by changes in cellular morphology, the buildup of metabolites or cell death and the accumulation of cell debris. This demonstrates the limitations of monitoring bioprocesses using OD without additional supporting measurements.

CO_2_ evolution is common in many metabolic pathways, making it a more direct measure of metabolism. During the mixed acid fermentation of *E*. *coli*, CO_2_ and H_2_ evolve from the decomposition of formate (see scheme in Fig. 1). Figure 6a shows the accumulated CO_2_ partial pressure in the closed system as measured by FTIR spectroscopy. While OD, pH, and other metabolites remain virtually constant during the lag phase (0–3 h), the CO_2_ rises immediately but slowly due to continued metabolic activity. Although there is no apparent cell division occurring during the lag phase, the inoculated cells will adapt from the shock of the transfer to a new medium as they synthesise the necessary enzymes required for metabolism in the new environment. M9 medium lacks essential amino acids and peptides found in more complex media (such as LB) and requires new enzymes to synthesise these biomass precursor molecules from oxaloacetate, malate, and glycolytic intermediates. At 3 h when exponential bacterial growth begins, the CO_2_ evolution rate increases and then continues into the stationary phase B in an S-shape curve. CO_2_ peaks and then plateaus at 78 mbar after 40 h during phase C indicating the end of fermentation.

In Fig. 6d, the FTIR measurements for acetaldehyde and ethanol show production starts at around 5 h as the lag phase ends. The intermediate acetaldehyde peaks at 18 ppm (corresponding to a concentration of 0.11 mM in solution using Henry’s law) at the end of phase A and then declines during phase B as it is converted to ethanol. Ethanol rapidly rises to 105 ppm (9 mM) in phase A and then trends upwards gradually to 135 ppm (12 mM) in phase B (with some minor ‘waves’ which we cannot explain at present). The increase in both volatiles during the exponential part of phase A appears to follow first-order kinetics with a rise time of *t*_1/2_ = 2.5 h. The disappearance of acetaldehyde in phase B at first has a slower decay with *t*_1/2_ = 30 h between 15 and 29 h, followed by a faster decay with *t*_1/2_ = 5.3 h. In control measurements, we have also sampled bacterial suspensions at different stages and measured the ethanol content by GC (solid symbols in Fig. 6d). We have found excellent agreement between FTIR and GC measurements corroborating our method, but note that our GC data is much more scattered.

Alongside CO_2_, acetaldehyde and ethanol monitoring by headspace FTIR spectroscopy, acetate and formate production can be monitored on-line by liquid-phase Raman spectroscopy. They both start at the end of the lag phase after 4 h and rise rapidly in the exponential phase to 20 mM acetate and 15 mM formate at 12 h (see Fig. 6c). In the following stationary phase B, formate then completely disappears almost linearly, indicating zeroth-order kinetics with rate constant 0.66 mM h^−1^ or 0.04 mmol h^−1^. At the same time, acetate shows only a tiny further increase to about 23 mM. Since CO_2_ production is observed as formate accumulates in phase A, it suggests that both production and consumption of formate are occurring here with a net increase of extracellular formate. This is corroborated by the 29 mM total of acetate, acetaldehyde, and ethanol produced in phase A being higher than the 15 mM formate produced. Formate is cleaved from pyruvate by pyruvate formatelyase to form acetyl-CoA (see Fig. 1) which will be ultimately converted to acetate or ethanol or enter the TCA cycle; thus, a minimum of 29 mM formate must have been produced in phase A. The discrepancy of 14 mM must be formate that was decomposed to CO_2_ and H_2_, which would correspond to 37.5 mbar of CO_2_ gas. However, only 21.5 mbar CO_2_ is observed in phase A, suggesting that in phase A, CO_2_ is also produced and consumed (with a net increase), likely due to the incorporation of carbonate during PEP conversion to oxaloacetate. Oxaloacetate and malate can be used as precursors for biosynthesis [1]; thus, oxaloacetate production will be essential during the exponential phase when the majority of the biomass is synthesised. In phase B, unlike phase A, there is a net decrease in formate due to its continued consumption overpowering its production. In phase B, only 3 mM acetate and 3 mM ethanol are produced, meaning at least 6 mM formate is produced and consumed alongside the 15 mM formate that accumulated in phase A. A total of 21 mM formate would correspond to 56 mbar CO_2_ in the headspace in good agreement with the 56.5 mbar of CO_2_ observed in phase B. This suggests that PEP conversion to oxaloacetate pathway is not as significant during the stationary phase as it is during exponential growth. We have confirmed the validity of the Raman measurements by comparing with acetate GC measurements of selected samples (solid symbols in Fig. 6c); the agreement between the different methods is satisfactory, but again, note the larger scatter of GC data.

From the concentrations of phosphate buffer anions also determined by liquid Raman analysis (see Fig. 6b), the pH and concentrations of net acid produced can be calculated. The spectroscopically determined pH starts at 7.15, which was confirmed by sampling the culture at the start and measuring the pH to be 7.1 using a pH meter (see Fig. 6b). The pH stays constant during the lag phase until 4 h. In the following exponential growth phase, the pH rapidly decreases to 5.5 due to the accumulating acids. A pH of 5.5 has been reported as significant for increasing H_2_ production compared with neutral pH due to increased expression of the *hyc* operon (encoding hydrogenase 3 in the FHL complex) [48]. During phase B, the pH recovers to a less acidic pH of 5.9 at 40 h and then remains constant during phase C. The end-point pH has been confirmed by a sample measured externally to have pH 5.9. The change in pH also directly reflects the concentration of net acid excreted into the medium by the bacterial metabolism (see Fig. 6c) which rises from around 4 h to peak at 52 mM at the end of the exponential phase at 14 h. This value essentially corresponds to the sum of acids formed as no basic products are expected. Each unit of acetate, formate, and lactate will provide roughly one acidic proton when the pH is between 5.5 and 7.1 based on their p*K*_a_ values of 4.76, 3.75, and 3.86, respectively. Succinate, however, being a dicarboxylic acid with p*K*_a1_ = 4.2 and p*K*_a2_ = 5.6, will produce up to 2 units of H^+^, depending on the pH. Of the 52 mM acid produced during phase A, 35 mM belongs to acetate and formate, and the remaining 17 mM likely originates from lactate and succinate. After 14 h in phase B, the total acid concentration declines by 10 to 42 mM at 40 h. The decline is due to the consumption of formate being higher than the total of the other acids produced during phase B. The recovery in pH during the stationary phase B shows how FHL complex activity is critical for preventing acidification of the medium.

The carbon balance during the mixed acid fermentation can be mostly accounted for. The initial 30 mM glucose (1.83 mmol of C_6_ glucose) provided 11 mmol of carbon atoms. At the end, CO_2_, acetate, and ethanol contained 1.7, 2.8, and 1.4 mmol of carbon atoms for a running total of 5.9 mmol. The dry biomass at the end was typically 30 mg, of which 48% can be assumed to be carbon by mass [49] which corresponds to 1.2 mmol of carbon atoms, increasing the running total to 7.1 mmol. The concentrations of lactate and succinate are unknown, but at the end, there are 19 mM of the 42 mM net acid concentration that is unaccounted for by the 23 mM acetate. As an estimation, by assuming this remaining acid is lactate, it would correspond to 3.5 mmol of carbon for a final total of 10.6 mmol. 10.6 mmol of carbon atoms at the end is very close to the initial 11 mmol provided by glucose which suggests that the glucose supply was depleted by the end of fermentation. Nearing glucose limitation may have influenced the transition to stationary phase growth at 12 h as most of the carbon balance can be accounted for at this time with only 3 mM acetate and 3 mM ethanol produced in phase B, corresponding to 0.7 mmol of carbon atoms.

In order to investigate how feeding a bioreactor with formic acid or formate will change the kinetics of formate consumption, and also as a pilot to investigate possible future isotope labelling studies, we injected 40 mM formic acid or potassium formate after the initial 3 h at the end of the lag phase. Formic acid addition was not successful as it killed the low bacterial density culture as no activity was observed after the addition. *E*. *coli* maintains a cytoplasmic pH of around 7.5, and as a result, organic acids exist in the dissociated form instead of the membrane-permeable acid form. Formate anions (p*K*_a_ = 3.75) are transported across the cytoplasmic membrane by the protein FocA (formate channel) [6, 50]. Other, as of yet unidentified, pH-dependent formate transport pathways exist as *E*. *coli* mutants lacking FocA can export 50% the level of formate exported by the wild type [6, 51]. External, undissociated formic acid can permeate the cell membrane and dissociate within the more alkaline cytoplasm, which increases the proton concentration and depresses the internal pH [5, 52]. An acidic cytoplasm negatively influences cell viability by affecting the integrity of purine bases and inducing denaturation of essential enzymes [53]. Acid anions can also negatively affect cell growth by causing an increase in potassium ion transport into the cell, leading to glutamate export and disruption of cytoplasm osmolarity [54].

**Fig. 7:**
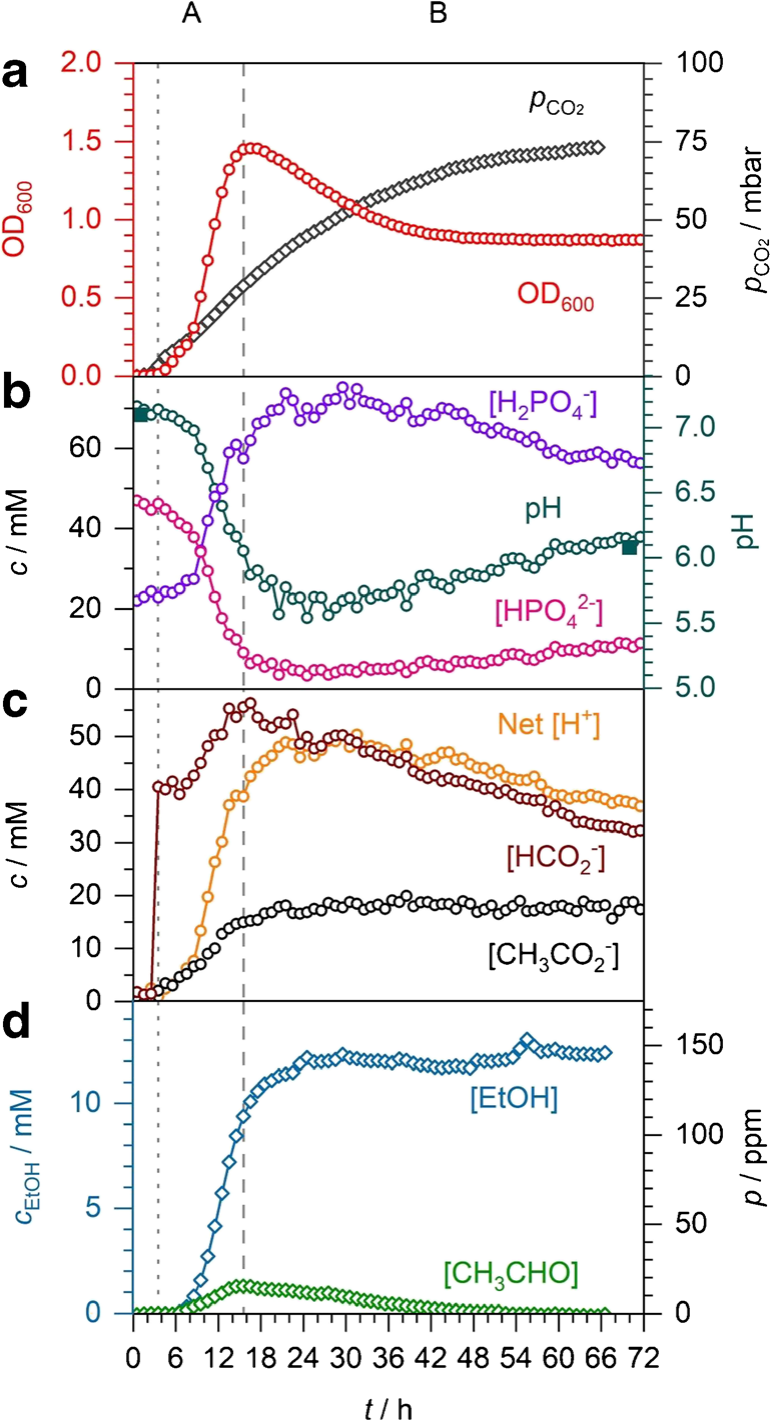
*E*. *coli* anaerobic fermentation in M9 medium with 40 mM potassium formate added at 3 h as shown by a dotted line. Phases A and B denote formate accumulation and decline, respectively. **a** OD of the bacterial culture and partial pressure of CO_2_. **b** Concentrations of the phosphate buffer components with in situ, spectroscopic pH (open circles) and externally measured pH (solid squares). **c** Concentrations of acetate, formate, and net acid produced as quantified by Raman spectroscopy. **d** Partial pressures of ethanol and acetaldehyde determined by FTIR spectroscopy with calculated dissolved ethanol concentrations

This problem was avoided by using the potassium formate salt, which is not acidic. Figure 7 shows anaerobic mixed acid fermentation in the closed system, similar to the experiment displayed in Fig. 6, but now with 40 mM potassium formate added at 3 h. The behaviour of the OD, CO_2_, acetaldehyde and ethanol partial pressures, phosphates, acetate concentrations, and the pH are similar to previous experiments and will not be discussed in detail. Of note, in Fig. 7, production of fermentation products is slightly slower compared with that in Fig. 6, suggesting that exogenous formate has an adverse effect on growth. The formate concentration remains unchanged until 7 h; however, acetate production begins immediately, indicating that no net formate is accumulating up to 7 h. From 7 to 16 h, 15 mM of formate accumulates, similar to Fig. 6, for a total of 55 mM. During phase B, net formate consumption occurs, again almost linearly until the end of the experiment. This suggests that formate consumption follows zeroth-order kinetics, with rate constant 0.42 mM h^−1^ or 0.025 mmol h^−1^, somewhat slower than in fermentation without formate addition (compare 0.66 mM h^−1^ or 0.04 mmol h^−1^). If formate consumption followed first or higher order kinetics, a faster decay after formate addition would be expected. A zeroth-order kinetic rate law could indicate high substrate saturation of the enzymes reaching the maximum rate of conversion or the rate of formate transport into the cell being rate-limiting. Based on this observation, we expect that addition of exogenous formate to a bioreactor is unlikely to speed up the rate of biohydrogen production from *E*. *coli* as the process appears to be limited by either FHL enzyme turnover or formate transport.

## Conclusions

We introduced an experimental setup for continuous monitoring of bacterial fermentation processes by simultaneous OD measurements, long-path FTIR gas monitoring, and liquid Raman and spectroscopic pH determination. Analysis of FTIR spectra provides partial pressures of CO_2_, ethanol, and acetaldehyde which can be converted to concentration in the bacterial suspension via Henry’s law. Liquid Raman spectroscopy provides concentrations of liquid and dissolved species, including formate and acetate anions and the anions of the phosphate buffer. Via a modified Henderson-Hasselbalch equation, this allows the spectroscopic, in situ determination of the pH of the suspension in the 6–8 pH range with an accuracy better than 0.1 and the calculation of the total net yield of acids. Our techniques are non-invasive, provide concentrations in real time, and do not require sampling, in contrast to standard analytical methods such as GC or mass spectrometry (MS). This allows measurements in closed systems or the measurement of the outgoing stream of a bioreactor for process control, for example. Our liquid Raman setup is truly contactless, just shining and collecting light through the glass capillary where the suspension is flowing. This is in contrast to commercial Raman probes which have to be immersed in the solution, which have issues with maintaining optical performance and sterile conditions. A final advantage of our spectroscopic approach is that it can be easily adapted to monitor additional components, its high selectivity due to unique spectroscopic signatures, the distinction of isotopomers which is essential for isotope labelling, and the ability to measure pH contactless. Spectroscopy is also a very cost efficient alternative to more expensive instruments such as GC and MS. We applied these techniques to study the mixed acid fermentation of *E*. *coli*. Different phases of bacterial growth have been observed and characterised, and we discussed the production of CO_2_, ethanol, and acetaldehyde as its precursor, and acids such as formate and acetate and the changes in pH in the context of the mixed acid fermentation pathway. Formate decomposition into CO_2_ and H_2_ is found to be governed by a zeroth-order kinetic rate law, showing that addition of exogenous formate to a bioreactor with *E*. *coli* is not expected to increase the rate of decomposition of formate or biohydrogen production.

In future work, we plan to extend our spectroscopic approach to monitoring of bioprocesses, looking for spectral features of other species such as lactate or glucose. We would also like to take advantage of the spectral distinction of isotopomers to perform isotope labelling studies to elucidate reaction mechanisms. In future work, we want to include our recently introduced technique of cavity-enhanced Raman spectroscopy [11, 20, 33, 34] to monitor gas-phase species by Raman spectroscopy, including H_2_. In conclusion, spectroscopic monitoring of bioprocesses has great potential to supplement or replace traditional sampling techniques.

## Electronic supplementary material

ESM 1 (PDF 923 kb)
