# Supplementary material for: On-line analysis and in situ pH monitoring of mixed acid fermentation by Escherichia coli using combined FTIR and Raman techniques
Source: Anal Bioanal Chem. 2020 Aug 14;412(26):7307–19. doi: 10.1007/s00216-020-02865-5 (PMC7497492; doi:10.1007/s00216-020-02865-5)
Supplement: Supplementary file 1 — (PDF 923 kb) [file 216_2020_2865_MOESM1_ESM.pdf]

Analytical and Bioanalytical Chemistry

Electronic Supplementary Material

On-line analysis and *in situ* pH monitoring of mixed acid fermentation by *Escherichia coli* using combined FTIR and Raman techniques

George D. Metcalfe, Thomas W. Smith, Michael Hippler

Content

S.1. Experimental Details of the Home-built Raman Spectrometer

S.2. Calibration Plots
- S.2.1. Liquid Raman Spectroscopy
- S.2.2. Gas Chromatography
- S.2.3. Gas Phase FTIR Spectroscopy with White cell

S.1. Experimental Details of the Home-built Raman Spectrometer

The home-built Raman spectrometer was first described in ref. [S1] and modified later as described in ref. [S2]; key components of the monochromator and the camera have been described in refs [S3,S4]. Briefly, a frequency doubled Nd:YAG laser, 532.2 nm, 20 mW (Lasos, GL3dT) emits green excitation light that is turned by 90° by a small mirror and coupled into a microscope objective. The small mirror was a 2 mm × 3 mm oval film deposited in the centre of a glass slide so as not to take away too much of the Raman backscattered light. The microscope objective is a 20x, 0.50 NA achromatic objective (OptoSigma, 028-0220) with a large clear aperture (8.2 mm). The objective focused the laser light very tightly at 2 mm distance from the objective front into the glass tube, as well as collimating the resulting Raman backscattered light. The sample volume is essentially the focus volume with an estimated spatial resolution below 100 μm. The backscattered light passed through the glass slide and was coupled into a lens and transmitted to the monochromator (Shamrock SR-750-A) equipped with 1200 l/mm grating, 750 nm blaze, and CCD camera (Andor i-Dus DU420A-OE at –80 °C). The grating provided a 880 cm^{-1} spectral range at about 0.8 cm^{-1} resolution. After wavenumber calibration, Raman peak position accuracy is estimated to be ± 3 cm^{-1}. Raman reference spectra were obtained in borosilicate NMR test tubes. A scheme of the Raman setup is part of Fig. 2 (main text). In addition, see below for two photos of the Raman spectrometer.

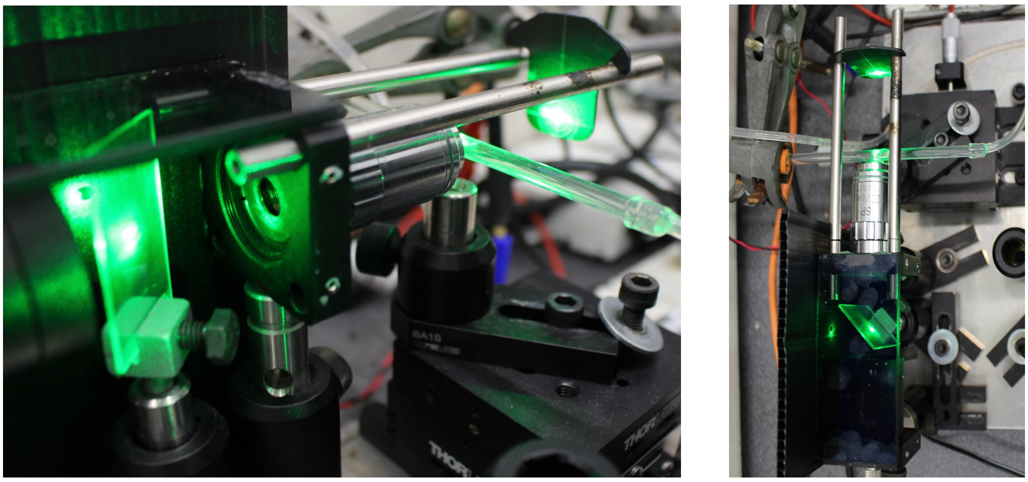

Fig. S1 Photographs of the home-built Raman spectrometer set-up, showing the laser beam path through the mirror and the microscope objective to the sample. Left: side view; right: top view

[S1] Mohr, C.; Spencer, C. L.; Hippler, M. Inexpensive Raman spectrometer for undergraduate and graduate experiments and research. *J. Chem. Educ.* **2010**, *87*, 326–330.

[S2] Ryabenkova, Y.; Jadav, N.; Conte, M.; Hippler, M.; Reeves-McLaren, N.; Coates, P. D.; Twigg, P.; Paradkar, A. Mechanism of Hydrogen-Bonded Complex Formation between Ibuprofen and Nanocrystalline Hydroxyapatite. *Langmuir* **2017**, *33*, 2965–2976.

[S3] Salter, R.; Chu, J.; Hippler, M. Cavity-enhanced Raman spectroscopy with optical feedback cw diode lasers for gas phase analysis and spectroscopy. *Analyst* **2012**, *137*, 4669–4676.

[S4] Hippler, M. Cavity-Enhanced Raman Spectroscopy of Natural Gas with Optical Feedback cw-Diode Lasers. *Anal. Chem.* **2015**, *87*, 7803–7809.

S.2. Calibration Plots

S.2.1. Liquid Raman Spectroscopy

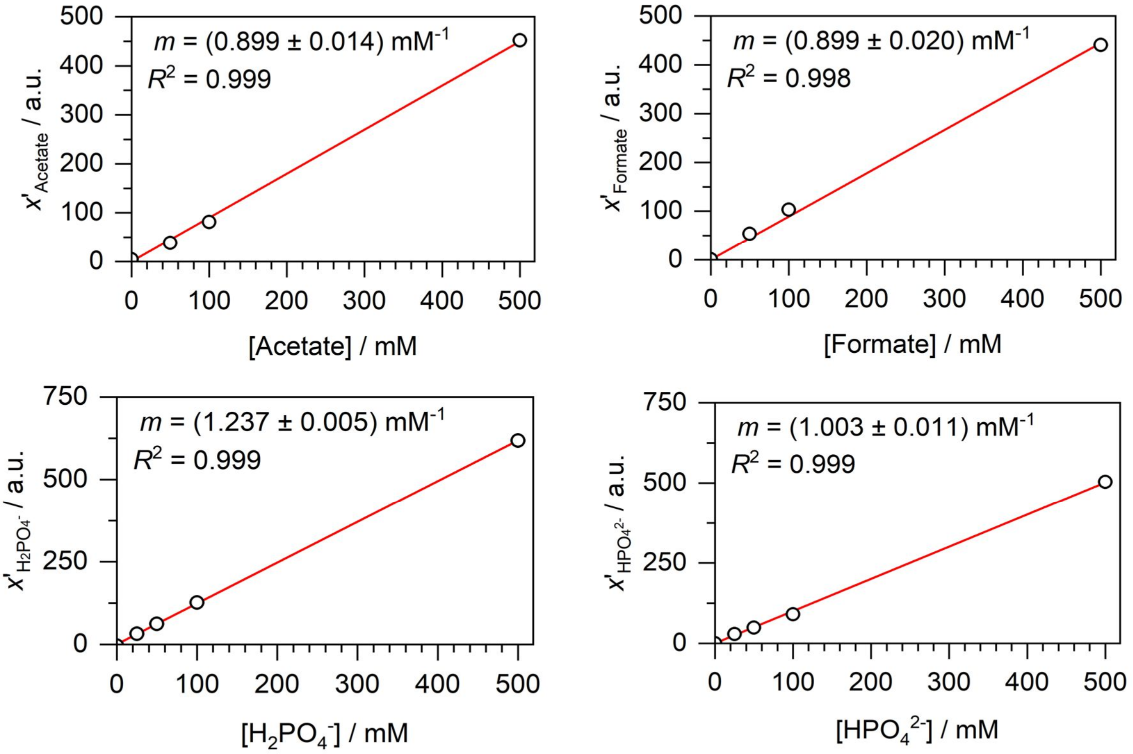

Fig. S2 Calibration plots of normalized Raman signals *x*' (in a.u.) *versus* concentration of anions in solution, including linear fit lines with slopes *m* and R^2 values

As described in the main text, experimental liquid Raman spectra were fitted with the sum of model Raman spectra of pure compounds of known concentration and a linear baseline (details given in the main text). The returned multipliers *x* were normalised (divided) by the water area peak (bending vibration of the water solvent at 1630 cm^{-1}) to give *x*'; this normalisation is particularly relevant for our biological samples which become turbid with time. In the normalisation, the water peak is fitted by a Gaussian contour centered at 1630 cm^{-1} with FWHM of 80 cm^{-1}. Normalisation assumes that the area of this Gaussian is the same in all solution Raman spectra because water concentrations remain the same. The normalized *x*' provide the concentration of the compound in comparison with the known concentration of the pure compound used as model in the fit. This procedure was validated by calibration plots shown below where the concentrations of calibration solutions were determined as described above and compared with the nominal concentrations. Excellent linearity (as shown by the R^2 value) and a good dynamic range are demonstrated in all cases. *m* denotes the slope of the calibration curves. Error bars, as represented by the standard deviation of repeat measurements, are approximately the size of the symbols used or smaller and are therefore not included in the calibration plots.

S.2.2. Gas Chromatography

For reference, concentrations of ethanol and acetic acid have also been measured by gas chromatography. 0.2 mL of sample was dissolved in 0.8 mL acetone, and 0.3 μL of this solution injected into a standard GC instrument (temperature programmed Agilent DB-WAX UI, with 1.4 mL/min H_2 carrier gas and FID detector). Retention times for ethanol and acetic acid were 4.5 and 11 minutes, respectively. From the GC peak integral, the concentration of the sample was determined after a calibration. The calibration plots are shown below, demonstrating good linearity and dynamic range. Error bars, as represented by the standard deviation of repeat measurements, are approximately the size of the symbols used or smaller and are therefore not included in the calibration plots.

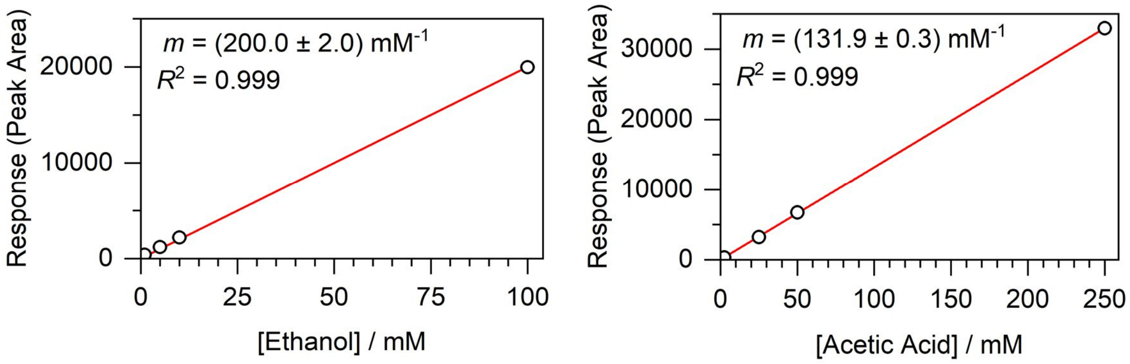

Fig. S3 Calibration plots for ethanol and acetic acid GC measurements

S.2.3. Gas Phase FTIR Spectroscopy with White cell

To determine gas phase concentrations (partial pressures), we applied the Beer-Lambert law with absorption cross sections from literature databases (HITRAN [S5] or PNNL [S6]), and compared integrated absorbances (band integrals) in the ranges specified in the main text. The spectral features (overtones, combination bands) were chosen to have absorbances well below ln $(I_0/I) < 2$, to avoid saturation effects. We validated this approach using N_2O as a test gas with the White cell set at 8 m absorption path length. The first calibration plot shows calculated band integrals using the known partial pressures and 8 m path length vs experimental integrated absorbance of the $2\nu_1$ overtone of N_2O (centred at 2563.5 cm^{-1}). For each filling pressure, a simulated absorption spectrum was generated assuming an 8 m path length. The figure below shows the measured $2\nu_1$ band integral for each filling pressure against the integrals for the same band in simulated spectra generated using cross-sections from HITRAN and assuming an 8 m absorption path length. The gradient of the plot is 0.995 ± 0.009, indicating that the predicted and measured spectra show very good agreement and that the White cell path length must be close to 8 m, as predicted by counting the total number of reflections of the HeNe alignment laser. Excellent linearity and 1:1 correspondence is seen. Error bars are approximately the size of the symbols used.

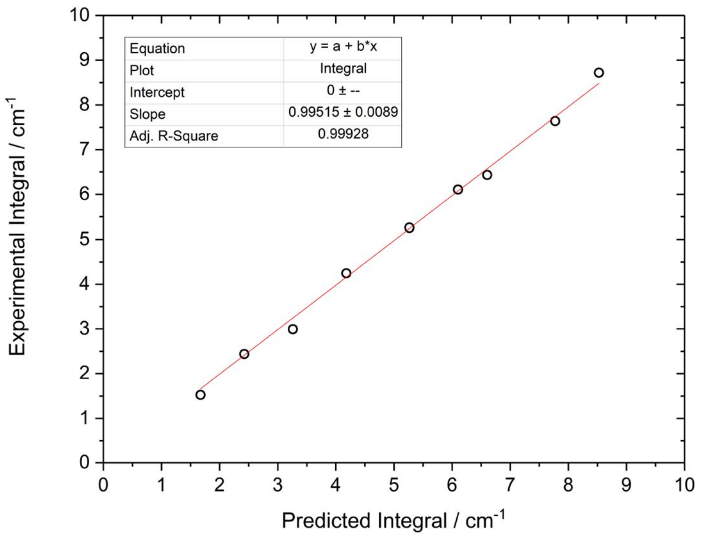

Fig. S4 Measured $2v_1$ overtone band integral for different filling pressure of N_2O against the integrals for the same band in simulated spectra generated using cross-sections from HITRAN and assuming an 8 m absorption path length

In a further calibration plot to corroborate our procedure, the figure below shows a nine point calibration based on the integral of the N_2O v_1+2v_2 combination band. As can be seen, within this pressure range, the band shows excellent linearity with increasing N_2O filling pressure.

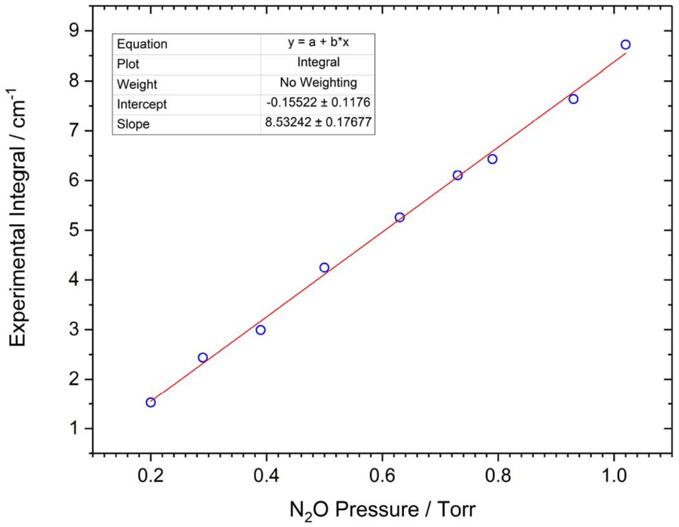

Fig. S5 Calibration based on the experimental integral of the N_2O v_1+2v_2 combination band versus N_2O filling pressure

[S5] Gordon, I. E.; Rothman, L. S.; Hill, C.; Kochanov, R. V.; Tan, Y.; Bernath, P. F.; Birk, M.; Boudon, V.; Campargue, A.; Chance, K. V.; Drouin, B. J.; Flaud, J.-M.; Gamache, R. R.; Hodges, J. T.; Jacquemart, D.; Perevalov, V. I.; Perrin, A.; Shine, K. P.; Smith, M.-A. H.; Tennyson, J.; Toon, G. C.; Tran, H.; Tyuterev, V. G.; Barbe, A.; Császár, A. G.; Devi, V.M.; Furtenbacher, T.; Harrison, J. J.; Hartmann, J.-M.; Jolly, A.; Johnson, T. J.; Karman, T.; Kleiner, I.; Kyuberis, A. A.; Loos, J.; Lyulin, O. M.; Massie, S. T.; Mikhailenko, S. N.; Moazzen-Ahmadi, N.; Müller, H. S. P.; Naumenko, O. V.; Nikitin, A. V.; Polyansky, O. L.; Rey, M.; Rotger, M.; Sharpe, S. W.; Sung, K.; Starikova, E.; Tashkun, S. A.; Vander Auwera, J.; Wagner, G.; Wilzewski, J.; Wcisło, P.; Yu, S.; Zak, E. J. The HITRAN2016 Molecular Spectroscopic Database. *J. Quant. Spectrosc. Radiat. Transf.* 2017, *203*, 3-69.

[S6] Sharpe SW, Johnson TJ, Sams RL, Chu PM, Rhoderick GC, Johnson PA. Gas-phase databases for quantitative infrared spectroscopy. Appl. Spectrosc. 2004;58:1452–1461.
